# The Relationships of Workplace Spirituality and Psychological Capital with Work Engagement Among Junior High School Teachers

**DOI:** 10.3390/bs15010044

**Published:** 2025-01-04

**Authors:** Shwu-Ming Wu

**Affiliations:** Department of Human Resource Development, National Kaohsiung University of Science and Technology, Kaohsiung 824004, Taiwan; mingwu@nkust.edu.tw

**Keywords:** workplace spirituality, psychological capital, work engagement, junior high school teachers

## Abstract

Engaged teachers demonstrate high levels of motivation and commitment to their work, increasing the likelihood of job retention and enabling them to perform at their best, ultimately benefiting both schools and students. Teacher engagement may be fostered by enhancing workplace spirituality and psychological capital. This study aimed to examine the relationships between and effects of workplace spirituality and psychological capital on work engagement, while also comparing demographic differences affecting these variables. A sample of 123 teachers was recruited from various junior high schools in Taiwan. The measured variables were workplace spirituality (including meaningful work, inner life, and sense of community), psychological capital (covering efficacy, hope, optimism, and resilience), and work engagement (comprising vigor, dedication, and absorption). The results indicate that male teachers exhibited higher levels of hope and overall psychological capital, while older teachers displayed greater optimism and a stronger sense of community. Additionally, strong positive correlations were found among workplace spirituality, psychological capital, and work engagement. Particularly, workplace spirituality and psychological capital were identified as significant predictors of work engagement among junior high school teachers. The implication for school organizations is that enhancing workplace spirituality and psychological capital should be prioritized as strategies to promote work engagement among junior high school teachers.

## 1. Introduction

Teachers’ work engagement is a crucial factor in the success of school organizations. Work engagement represents a positive psychological state in which individuals are emotionally, cognitively, and physically involved in their work, approaching tasks with enthusiasm and energy (Meng et al., 2022). It is also a motivational concept that involves willingly dedicating personal resources to meet the demands of tasks associated with a particular vocational role (Christian et al., 2011). Job resources possess motivational potential and lead to positive organizational outcomes through work engagement (Mazzetti et al., 2023). Work engagement has become an important focus in many organizations due to its strong association with employee well-being and performance (Christian et al., 2011; Halbesleben, 2010). It is considered a unique and significant personal and organizational construct contributing to both well-being and thriving at work (Hirschi, 2012; Xanthopoulou et al., 2012). Engagement encompasses involvement, commitment, passion, enthusiasm, absorption, focused effort, zeal, immersion, dedication, and energy. Schaufeli et al. (2002) viewed engagement in the work setting as multidimensional so that it was termed work engagement. It is characterized by high levels of motivation and commitment. In the context of teaching, work engagement can manifest as a teacher’s passionate involvement in their work, driven by enthusiasm for teaching, a commitment to professional development, and a strong sense of responsibility for their students’ success. Studies have highlighted teachers who exhibit high levels of work engagement tend to be more effective, motivated, and satisfied in their teaching roles (Klassen et al., 2012). Employees with high levels of work engagement are able to convert the positive energy and inspiration gained from various activities into productive resources in the workplace (Bakker et al., 2020). Work engagement is closely linked to teachers’ attitudes toward their profession and involves efforts to increase their happiness, satisfaction, dedication, and commitment, which enables them to perform at their best for the benefit of students, parents, and society. Thus, teachers’ work engagement is a key factor in improving their overall performance.

In Taiwan, junior high school (Grades 7–9, ages 12–15) focuses on general education, foundational learning, and preparation for high school entrance exams, with greater emphasis on discipline. Senior high school (Grades 10–12, ages 15–18) offers specialized academic, vocational, or comprehensive tracks aimed at university preparation or career training, fostering more student autonomy. Junior high teachers face higher stress due to exam preparation and behavior management, while senior high teachers focus on subject specialization and student career guidance (Huang & Huang, 2019). Also, junior high school teachers today face increased stress and workloads compared to previous years (Huang & Huang, 2019). Engaged workers experience less stress and depression than their non-engaged counterparts (Mazzetti et al., 2023) and satisfied workers demonstrate high motivation and job commitment, which increases the likelihood of job retention (Salanova et al., 2011). Therefore, this study aims to explore work engagement among junior high school teachers.

Spirituality is recognized as a crucial factor influencing job satisfaction and performance in organizations (Kumpikaitė, 2014). Workplace spirituality is often linked to positive effects on job satisfaction (Bodia & Ali, 2012; Hassan et al., 2016; Kumpikaitė, 2014). It also plays a key role in helping individuals overcome the conflicts in their lives (Milliman et al., 2003) and inspiring employees to find fulfillment in their work life (Budagavi et al., 2021). de Klerk (2005) explored the spiritual framework, highlighting the importance of finding meaning in life and its contribution to enhancing work wellness. Since work engagement is characterized by cognitive absorption, scholars suggest that spirituality provides meaning, which facilitates employee engagement in their tasks (Saks, 2011; Yimprasert et al., 2019). The concept of meaningful work is central to workplace spirituality (Ashmos & Duchon, 2000; Hassan et al., 2016), and previous studies have shown that workplace spirituality significantly impacts work engagement (Baskar & Indradevi, 2020; Shuck et al., 2011; Yimprasert et al., 2019). Introducing spirituality into the workplace may lead to higher job satisfaction for teachers. The pursuit of meaning in work, as proposed by Osman-Gani et al. (2013) and Pawar (2009), has the potential to boost work engagement. Jasmeet and Chopra (2018) conducted a study with 275 full-time government employees in India, finding a positive correlation between components of workplace spirituality (inner life, meaningful work, and community) and components of work engagement (attention and absorption). Roof (2015) also found that spirituality in the workplace is related to employee engagement. Similarly, Mahipalan (2018) examined the connections among workplace spirituality, employee engagement, and teaching satisfaction among secondary school teachers, revealing significant positive associations. Owing to the novelty of the proposed study, this study aims to examine the relationship between workplace spirituality and work engagement among teachers.

Psychological capital has been conceptually and empirically demonstrated to be a core construct which can predict desired employee outcomes, such as performance and job satisfaction, better than the individual resources independently (Luthans et al., 2007). Psychological capital refers to an individual’s positive psychological state, characterized by efficacy, hope, optimism, and resilience (Luthans et al., 2007). It is seen as a personal resource that enhances well-being, performance, and the ability to cope with challenges in work and life. A study by Shabeeba and Manikandan (2019) revealed that dimensions of psychological capital, such as self-efficacy and optimism, along with emotional intelligence dimensions like emotional management, emotional awareness, socio-emotional awareness, and relationship management, can predict job performance.

Herdem (2019) explored the effect of the psychological capital of undergraduate students on their motivation and found that resilience in the psychological capital significantly and positively influenced motivation for achievement. Conceptualizing positive psychological capacities as accessible resources offers a crucial theoretical explanation for understanding how such positive capacities impact work engagement. Specifically, psychological resources derived from positive emotions experienced by employees may lead to attitudes like emotional engagement (Hodges, 2010), which is characterized by work engagement. Additionally, Herdem (2019) explored the influence of psychological capital on the motivation of undergraduate students, revealing resilience as a significant and positive impact on motivation for achievement. Moreover, Avey et al.’s (2008) study found that psychological capital was linked to positive emotions, which, in turn, were related to attitudes such as engagement. In other words, employees with positive psychological capital may contribute to high work engagement. Therefore, this study aims to explore the relationship between psychological capital and work engagement of teachers.

Moitreyee and Lalatendu (2022) found a positive relationship between workplace spirituality and teachers’ professional well-being, with psychological capital partially mediating this relationship. Bhattacharyya and Afroz (2023) demonstrated a positive relationship between psychological capital and workplace spirituality in predicting organizational citizenship behavior, and a negative relationship between psychological capital, workplace spirituality, and counterproductive work behavior. Additionally, workplace spirituality and psychological capital were found to influence happiness at work, with work engagement acting as a mediator (Thawinratna, 2023). While both workplace spirituality and psychological capital independently predict positive outcomes, their combined predictive value may be even stronger when considered together. Although previous studies have explored the relationship between psychological capital and work engagement in various organizational settings (Schaufeli et al., 2002), limited empirical research has specifically focused on the educational sector or junior high school teachers. Similarly, while the impact of workplace spirituality on employee attitudes and engagement has been studied in other contexts (Bodia & Ali, 2012; Roof, 2015), the specific relationship between workplace spirituality and teacher engagement in junior high schools remains underexplored. Hence, this study aims to explore the relationships and effects of workplace spirituality and psychological capital on work engagement.

Regarding the main variables of this study, demographic factors like gender, age, and education level have also shown some relevance. Male teachers often exhibit higher psychological capital, characterized by resilience and self-efficacy, while female teachers show stronger emotional engagement and workplace spirituality (Mahipalan, 2018). Older teachers, driven by experience and job security, report higher psychological capital as greater hope and resilience, whereas younger teachers display stronger workplace spirituality, fueled by idealism and purpose (Mahipalan, 2018). Engagement tends to increase with age, supported by role alignment and positive work environments. Higher education also enhances engagement and spirituality, fostering deeper connections to meaningful work (Hassan et al., 2016; Saks, 2011). Thus, this study aims to compare the demographic differences in junior high school teachers on workplace spirituality, psychological capital, and work engagement.

### 1.1. Theoretical Framework

#### 1.1.1. The Construct of Work Engagement

Work engagement is defined as a positive, fulfilling, work-related state of mind characterized by vigor, dedication, and absorption (Mazzetti et al., 2023; Schaufeli et al., 2002). Vigor refers to high levels of energy and mental resilience while working, a willingness to invest effort in one’s work, and persistence even in the face of difficulties. Dedication involves being intensely involved in work tasks and experiencing a sense of significance, enthusiasm, inspiration, pride, and challenge. Absorption is characterized by full concentration and a sense of being happily engrossed in one’s work. Another study stated that vigor and dedication are the core dimensions of engagement (Gonza’lez-Roma et al., 2006). Based on the study of Schaufeli et al. (2002), work engagement is identified as comprising three dimensions, vigor, dedication, and absorption.

#### 1.1.2. The Construct of Workplace Spirituality

With the growing interest in integrating spirituality into the workplace (Delbecq, 2005), the spiritual quest within positive psychology has emerged as a key issue. Workplace spirituality has been defined in various ways, including as a value and belief system, a developmental path, a way to connect with one’s inner self, a means for self-realization, and an inner experience (Garg, 2017). Spirituality, deeply intertwined with personal values such as love, affection, tolerance, satisfaction, responsibility, and harmonious relationships with oneself and others (Kaya, 2015), aligns closely with the principles of positive psychology. Karakas (2010) conducted a comprehensive review of 140 articles on workplace spirituality, revealing three distinct perspectives on how spirituality contributes to organizational performance. Workplace spirituality involves employees recognizing themselves as spiritual beings, requiring nourishment for their souls within the work context. It is also about experiencing a sense of purpose and meaning in their work and finding meaning in the performance of tasks (Rocha & Pinheiro, 2021) as well as experiencing a sense of interconnectedness and community. Then, workplace spirituality is expressed through employees’ experiences of meaning, purpose, community, and transcendence in their work (de Klerk, 2005; Prabhu et al., 2017). Milliman et al. (2003) conceptualized workplace spirituality as encompassing three key aspects, meaningful work, sense of community, and alignment with organizational values. Similarly, the Spirituality at Work instrument developed by Ashmos and Duchon (2000) identified three dimensions of workplace spirituality, inner life, meaningful work, and community. Drawing from Ashmos and Duchon (2000) and Milliman et al. (2003), this study measured workplace spirituality using three components, inner life, meaningful work, and sense of community.

#### 1.1.3. The Construct of Psychological Capital

The core construct of psychological capital, which comprises the positive psychological resources of efficacy, hope, optimism, and resilience, has been linked to various employee attitudes, behaviors, and performance outcomes (Avey et al., 2008; Avey et al., 2010). Employees facing organizational change need the efficacy to adapt and the resilience to recover from setbacks inherent in the change process. Successfully navigating change requires motivation, determination, and hope to overcome obstacles, as well as an optimistic outlook when challenges arise and a positive view of the future. Luthans and colleagues (Avey et al., 2010; Luthans et al., 2007; Luthans & Youssef-Morgan, 2004, 2017) have identified efficacy, hope, optimism, and resilience as key constructs that best meet the criteria for inclusion in what they term psychological capital. These four components have consistently aligned with the criteria defining positive organizational behavior (Avey et al., 2010; Avey et al., 2008; Luthans et al., 2007). Thus, in this study, psychological capital refers to an individual’s positive psychological state of development, characterized by efficacy, hope, optimism, and resilience.

#### 1.1.4. The Relationships of Workplace Spirituality and Psychological Capital with Work Engagement Among Teachers

Workplace spirituality, identified as a factor enhancing work engagement (van der Walt, 2018), serves as a key indicator of work engagement. Known for fostering positive work attitudes, behaviors, improved performance, and job satisfaction, workplace spirituality is likely to positively influence work engagement. Similarly, empirical evidence suggests that psychological capital contributes to better performance, positive work outcomes, and favorable workplace attitudes and behaviors (Gurbuz & Yildirim, 2019). Kotzé (2018) found that psychological capital has a positive influence on work engagement. On the other hand, junior high school teachers with higher levels of workplace spirituality and psychological capital are better equipped to navigate the complexities of teaching, curriculum management, and student interactions. As a result, they may demonstrate greater enthusiasm and engagement in their school-related responsibilities. A study by Obuobi-Donkor et al. (2022) highlights the significant impact of self-efficacy, engagement, and emotional resilience on teachers’ ability to manage stress and avoid depression. Teachers who demonstrated higher levels of self-efficacy, engagement, and emotional resilience were better able to cope with stress and reduce the risk of burnout and depression (Obuobi-Donkor et al., 2022). Thus, workplace spirituality and psychological capital are closely related to work engagement of teachers.

### 1.2. Purpose of Study and Hypotheses

Based on the above analyses, this study proposes to achieve the following objectives:

1. To compare the demographic variables (gender, age, education, year of working experience) of differences among junior high school teachers regarding workplace spirituality, psychological capital, and work engagement.
2. To examine the relationships among workplace spirituality, psychological capital, and work engagement.
3. To explore the impact of workplace spirituality and psychological capital on work engagement.

Based on the above purposes, the hypotheses of this study are as follows:

**Hypothesis** **1:** *There are significant demographic variables (gender, age, education, year of working experience) differences between teachers regarding workplace spirituality, psychological capital, and work engagement.*

**Hypothesis** **2:** *Workplace spirituality is significantly positively related to psychological capital.*

**Hypothesis** **3:** *Workplace spirituality is significantly positively related to work engagement.*

**Hypothesis** **4:** *Psychological capital is significantly positively related to work engagement.*

**Hypothesis** **5:** *Workplace spirituality and psychological capital have significant positive effects on work engagement.*

## 2. Material and Methods

### 2.1. Participants and Procedure

A sample of 123 teachers was recruited from 15 junior high schools in the southern regions of Taiwan between May and June 2024. The selection of the schools was based on a purposive sampling of the southern area. A questionnaire link was sent to the selected junior high school teachers, who were asked to invite other teachers to participate. Each school contributed approximately 5 to 10 teachers, resulting in a total of 123 teachers participating in the study. The sample consisted of 48 male and 75 female teachers, with ages ranging from 25 to 55 years and a mean age of 46.67. All participants voluntarily completed the survey. The frequency distribution of demographic variables is presented in Table 1.

### 2.2. Measures

In this study, three instruments were used to collect data from junior high school teachers, the Spirituality at Work Scale (SAWS), the Psychological Capital Questionnaire (PCQ), and the Utrecht Work Engagement Scale (UWES). These scales were chosen to measure workplace spirituality, psychological capital, and work engagement. Since the participants were Taiwanese teachers, the scales were translated from English to Chinese by the author. Two bilingual reviewers were then invited to verify the translation, and a few item wordings were adjusted based on their feedback.

#### 2.2.1. Spirituality at Work Scale (SAWS)

The SAWS, developed based on the studies by Ashmos and Duchon (2000) and Milliman et al. (2003), was employed to assess the experience of spirituality in the workplace. The measurement encompassed three aspects of meaningful work, inner life, and sense of community. All 18 item responses were scored on a 5-point scale. Although the original scale demonstrated high reliability and validity, differences in research subjects and culture remained. To assess the psychometric properties of the scale for this study, a pilot test with 100 teachers was conducted to evaluate its reliability and validity. An exploratory factor analysis of the 18 items was performed using the maximum likelihood method with Varimax rotation. The analysis revealed that all items loaded onto three factors with eigenvalues greater than unity, collectively explaining 68.72% of the total variance. Consequently, an 18-item scale was derived, encompassing the dimensions of meaningful work, inner life, and sense of community. Cronbach’s alpha values for these three subscales were 0.92, 0.83, and 0.91, respectively, while the overall scale demonstrated high internal consistency with a Cronbach alpha coefficient of 0.95.

#### 2.2.2. Psychological Capital Questionnaire (PCQ)

The PCQ was developed based on previous studies (Avey et al., 2010; Avey et al., 2008; Luthans et al., 2007). Following the conceptualization by Luthans et al. (2007), the scale measures psychological capital through four positive constructs, efficacy, hope, optimism, and resilience. All 13 responses are scored on 5-point scales, ranging from 1 (strongly disagree) to 5 (strongly agree), with higher scores representing higher psychological capital. While the original scale showed strong reliability and validity, variations in subjects and cultural context necessitated further evaluation. A pilot study with 100 teachers was conducted to reexamine the scale’s reliability and validity for this research. After an exploratory factor analysis of the 13 items, utilizing the maximum likelihood method with Varimax rotation, results showed that those items loading on four factors had the largest eigenvalues, accounting for 64.39% of the total variance. Consequently, a 13-item scale was derived, encompassing efficacy, hope, optimism, and resilience. Cronbach’s alpha values for these four subscales were 0.84, 0.87, 0.88, and 0.75, with an overall scale reliability coefficient of 0.92.

#### 2.2.3. Utrecht Work Engagement Scale (UWES)

The UWES, developed by Schaufeli et al. (2006), was designed to assess three dimensions, vigor, dedication, and absorption. All 9 item responses were rated on a 5-point scale. Despite the original scale’s high reliability and validity, differences in subjects and cultural context persisted. To verify the scale’s suitability for this study, a pilot test involving 100 teachers was carried out to reassess its reliability and validity. An exploratory factor analysis of the 9 items was conducted using the maximum likelihood method with Varimax rotation. The analysis revealed that all items loaded on three factors, each with eigenvalues greater than unity, collectively explaining 64.48% of the total variance. As a result, a 9-item scale was established, encompassing the dimensions of vigor, dedication, and absorption. Cronbach’s alpha values for these three subscales were 0.90, 0.88, and 0.82, with an overall scale reliability coefficient of 0.93.

### 2.3. Design and Analyses

The initial phase of this study involved the development, design, and implementation of three psychometric instruments aimed at measuring workplace spirituality, psychological capital, and work engagement. Cronbach α coefficients were employed, and the validity of the scales was examined using principal component analyses. Moreover, an Analysis of Variance (ANOVA) was used to compare demographic differences among teachers, and an F test was used to analyze gender differences. Subsequently, a predictive correlational design was adopted. Correlation matrices were computed to examine the relationships among workplace spirituality, psychological capital, and work engagement. Finally, a stepwise multiple regression analysis was employed to determine whether workplace spirituality and psychological capital served as significant predictors of work engagement.

## 3. Results

### 3.1. Demographic Variables of Differences Among Junior High School Teachers on Workplace Spirituality, Psychological Capital, and Work Engagement

An Analysis of Variance (ANOVA) was conducted to compare mean scores of demographic variables (gender, age, education, year of working experience) differences among teachers regarding their scores for workplace spirituality, psychological capital, and work engagement. The analysis revealed that only the variables of gender and age were statistically significant (*p* < 0.05). Since only gender and age displayed significant differences among teachers in terms of workplace spirituality and psychological capital, the means, standard deviations, and F tests for gender and age on these variables are presented in Table 2.

Gender showed significant differences in terms of hope and the total psychological capital score, while age displayed significant differences in terms of optimism and sense of community. Based on the mean scores, male teachers scored higher in hope and the total psychological capital scale compared to their female counterparts. Furthermore, the post hoc Scheffé test indicate that teachers aged over 51 demonstrated higher levels of optimism and a stronger sense of community than those in the 31–40 age group.

### 3.2. Correlations Among Workplace Spirituality, Psychological Capital, and Work Engagement

Correlational analyses were conducted to examine the relationships among workplace spirituality, psychological capital, and work engagement. The correlation between workplace spirituality and psychological capital is shown in Table 3, revealing a significant positive correlation between all dimensions of workplace spirituality and psychological capital. The correlations of workplace spirituality and psychological capital with work engagement are presented in Table 4. The results indicate that all dimensions of workplace spirituality and psychological capital were significantly positively correlated with work engagement. Thus, both workplace spirituality and psychological capital demonstrated a strong association with work engagement.

Stepwise multiple regression analyses were conducted to assess the predictive capacity of workplace spirituality and psychological capital on work engagement. First of all, to test multicollinearity in regression analysis, the variance inflation factor (VIF) for all the predictor variables ranged from 0.434 to 2.306. A VIF of 1 indicates no correlation among the predictors, while a VIF less than 5 suggests moderate correlation, which is generally acceptable. Based on these VIF values, there is an absence of multicollinearity.

Next, a stepwise multiple regression analysis was conducted to explore whether workplace spirituality and psychological capital were the best predictors of work engagement. Two types of criterion variables were entered separately. The first type of criterion variables included the three dimensions of work engagement (vigor, dedication, and absorption), with the predictors being the three dimensions of workplace spirituality (meaningful work, inner life, and sense of community) and the four dimensions of psychological capital (efficacy, hope, optimism, and resilience). The second type of criterion variable was the total scale of work engagement, with the predictors being the total scales of workplace spirituality and psychological capital. Since there were four criterion variables in total, four separate regression equations were analyzed. Table 5 presents the results of these four separate regression analyses. As summarized in Table 5, the results revealed that two dimensions of workplace spirituality, meaningful work and inner life, significantly predicted vigor, while meaningful work and sense of community significantly predicted dedication. Additionally, meaningful work (workplace spirituality) and efficacy (psychological capital) were significant predictors of absorption. Both the total scales of workplace spirituality and psychological capital were significant predictors of overall work engagement. Table 4 shows a strong relationship between workplace spirituality and psychological capital with work engagement. Consequently, both workplace spirituality and psychological capital emerged as strong predictors of work engagement.

## 4. Discussion

Regarding demographic variable differences, male teachers demonstrated higher hope and overall psychological capital compared to their female counterparts, while older teachers aged over 51 age exhibited greater optimism and stronger sense of community. The findings are partially consistent with Hypothesis 1, and the differences in psychological capital and workplace spirituality among teachers, with regard to gender and age, are supported by various studies. Male teachers often demonstrate higher psychological capital, including increased hope and overall resilience. This is consistent with findings from Wu (2024) and Mahipalan (2018), who noted that male teachers generally show greater efficacy and resilience in psychological capital compared to their female counterparts. Additionally, the significant differences in optimism and sense of community among teachers aged over 51 support the notion that older teachers tend to have a stronger connection to their workplace and exhibit greater optimism. One possible reason for these gender and age differences in psychological capital is the varying life experiences and coping mechanisms that male and older teachers may have developed over time. For instance, male teachers might report higher levels of hope because they may approach challenges with more confidence or have more years of experience handling stressors at work. Similarly, older teachers may possess a greater sense of community and optimism, possibly due to their accumulated experiences, which allow them to manage stressors better and develop stronger interpersonal connections. Moreover, given that male teachers expressed higher hope and psychological capital, and teachers aged over 51 demonstrated more optimism and stronger sense of community compared to their female counterparts and those within the 31–40 age range, it can be inferred that they may adapt more effectively to the school environment and find enjoyment in teaching even when confronted with increased work stress. Citing Obuobi-Donkor et al. (2022), it is asserted that teachers who exhibited greater self-efficacy, engagement, and emotional resilience were better able to manage stress and reduce depression, and experience better psychological health. Studies also show mixed results regarding the impact of education and work experience on psychological capital and workplace spirituality. Herdem (2019) found no significant effect of work experience on workplace spirituality. Similarly, Wu (2024) observed that while years of experience positively affected psychological capital dimensions like efficacy, it had minimal impact on workplace spirituality. Additionally, education level showed little effect on workplace spirituality or psychological capital. These findings highlight that age and gender may play a more significant role than education and work experience in shaping these constructs.

Moreover, consistent with Hypotheses 2–5, the study revealed highly positive correlations among workplace spirituality, psychological capital, and work engagement. Both workplace spirituality and psychological capital emerged as factors strongly related to work engagement and were identified as significant predictors of work engagement. The intense competition surrounding high school entrance examinations places a heavier workload and increased stress on junior high school teachers, potentially leading to lower work motivation and engagement. Pihie and Elias (2004) have indicated that the teachers cite factors such as workload, pressure, and unsatisfactory leadership for not favoring the teaching profession. Mousa (2020) discovered that workplace spirituality positively influences employee performance. Similarly, according to the findings of van der Walt (2018), workplace spirituality is linked to enhanced work engagement. In a study conducted by Jasmeet and Chopra (2018), positive correlations were identified between components of workplace spirituality (inner life, meaningful work, and community) and components of work engagement (attention and absorption). These insights collectively suggest that fostering workplace spirituality may play a pivotal role in improving work engagement, particularly in contexts marked by heightened competition and stress, such as the environment surrounding high school entrance examinations.

Sridevi and Srinivasan (2012) highlighted that psychological capital can contribute to improved performance, work outcomes, positive work attitudes, and behaviors. Also, Avey et al. (2008) discovered that psychological capital is linked to positive emotions, which, in turn, are associated with attitudes such as engagement. Dewi et al. (2021) indicated that work involvement or work engagement, work environment, and morale have a significant positive effect on teacher job satisfaction, emphasizing that motivated teachers are more likely to work toward enhancing their own satisfaction. Therefore, teachers’ work engagement is a critical factor in improving both teacher performance and the efficiency of educational organizations, as reflected in their enthusiasm, dedication, and job absorption (Bakker et al., 2014). Moreover, studies by Joo et al. (2016) and Gao et al. (2023) demonstrate that psychological capital significantly enhances teacher work engagement. Joo et al. (2016) found that teachers with higher levels of psychological capital, including self-efficacy, hope, and optimism, showed greater work enthusiasm. Similarly, Gao et al. (2023) identified that teachers with higher psychological capital reported higher work engagement. Both studies supported the findings that teachers with higher psychological capital demonstrate greater work engagement.

In summary, both workplace spirituality and psychological capital were the significant predictors of work engagement. The findings suggest that teachers who display higher workplace spirituality and psychological capital are more likely to experience greater work engagement. Increased workplace spirituality and enhanced psychological capital are associated with higher levels of teacher work engagement, consistent with previous findings (Avey et al., 2008; Jasmeet & Chopra, 2018; Luthans & Youssef-Morgan, 2017; van der Walt, 2018). Recognizing the significance of enriching workplace spirituality and strengthening teachers’ psychological capital can lead to improved work engagement. Overall, these results highlight the importance of promoting workplace spirituality and psychological capital to enhance teacher work engagement.

## 5. Conclusions and Implication

Male teachers exhibited higher hope and overall psychological capital, while those aged over 51 teachers demonstrated higher optimism and sense of community. The implication for female teachers is to focus on promoting psychological capital, while younger teachers should strive to enhance both psychological capital and workplace spirituality. This proactive approach is suggested to contribute to their ability to navigate the challenges of teaching and potentially foster a more positive and fulfilling professional experience.

Then, both workplace spirituality and psychological capital were the significant predictors of work engagement. The implication for junior high school organizations is that they should foster spiritually based cultures to enhance workplace spirituality and cultivate a more positive psychological capital environment, which, in turn, is expected to encourage greater work engagement among teachers, ultimately contributing to the overall improvement of education quality within their schools.

Overall, the findings of this study contribute to a better understanding of the relationships among workplace spirituality, psychological capital, and work engagement. They also provide insights into developing effective strategies to improve teacher work engagement in schools, ultimately enhancing school competitiveness. This study, which involved 123 junior high school teachers in southern Taiwan, suggests that future research could strengthen external validity by expanding the sample size. While the study highlights the positive impact of workplace spirituality and psychological capital on work engagement, further research could investigate additional factors influencing teacher engagement, such as organizational support, school culture, principal leadership, psychological climate, and other aspects. 

## Figures and Tables

**Table 1:** Frequency Distribution of Demographic Variables.

Demographic Variables	Categories	Frequencies	%
Gender	1. Male	48	39
	2. Female	175	61
Age	1. Below 30	9	7.3
	2. 31–40	19	15.4
	3. 41–50	52	42.3
	4. Above 51	43	35.0
Education	1. Teacher Training College	20	16.3
	2. General University	29	23.6
	3. Graduate School and Above	74	60.2
Years of Working	1. 1–5	10	8.1
	2. 6–10	14	11.4.
	3. 11–15	10	8.1
	4. More than 16	89	72.4

Note. N = 123.

**Table 2:** Means, Standard Deviation, and F Tests for Gender and Age regarding Workplace Spirituality and Psychological Capital.

Variables	Demographic Variables	M	SD	F	Scheffé
	Gender				
Hope	(1) Male	17.10	2.26	4.14 *	
	(2) Female	15.99	2.59		
Total Scale of Psychological Capital	(1) Male	54.98.	7.15	4.29 *	
	(2) Female	51.39	8.01		
	Age				
Optimism	(1) Below 30	10.00	4.41	3.52 *	(4) > (2)
	(2) 31–40	10.16	3.06		
	(3) 41–50	11.65	2.44		
	(4) Above 51	12.60	1.81		
Sense of Community	(1) Below 30	29.89	4.89	3.96 **	(4) > (2)
	(2) 31–40	26.84	6.54		
	(3) 41–50	30.75	5.44		
	(4) Above 51	31.28	5.72		

Note. N = 123. * *p* < 0.05; ** *p* < 0.01.

**Table 3:** Correlations between Workplace Spirituality and Psychological Capital.

Variables	Meaningful Work	Inner Life	Sense of Community	Total Scale of Workplace Spirituality
Efficacy	0.56 **	0.49 **	0.39 **	0.52 **
Hope	0.75 **	0.72 **	0.47 **	0.69 **
Optimism	0.66 **	0.98 **	0.51 **	0.66 **
Resilience	0.66 **	0.72 **	0.48 **	0.65 **
Total Scale of Psychological Capital	0.78 **	0.88 **	0.55 **	0.75 **

Note. N = 123. ** *p* < 0.01.

**Table 4:** Correlations of Workplace Spirituality and Psychological Capital with Work Engagement.

Variables	Vigor	Dedication	Absorption	Total Scale of Work Engagement
Efficacy	0.53 **	0.52 **	0.49 **	0.57 **
Hope	0.73 **	0.67 **	0.56 **	0.73 **
Optimism	0.69 **	0.54 **	0.50 **	0.65 **
Resilience	0.63 **	0.58 **	0.50 **	0.64 **
Total Scale of Psychological Capital	0.77 **	0.69 **	0.61 **	0.77 **
Meaningful Work	0.83 **	0.80 **	0.68 **	0.86 **
Inner Life	0.73 **	0.61 **	0.54 **	0.71 **
Sense of Community	0.51 **	0.62 **	0.41 **	0.57 **
Total Scale of Workplace Spirituality	0.73 **	0.78 **	0.63 **	0.80 **

Note. N = 123. ** *p* < 0.01.

**Table 5:** Summary of stepwise multiple regression for workplace spirituality and psychological capital predicting work engagement.

Criterion Variables	Predictor Variables	Standardized Beta	Multiple R	Multiple R^2^	Increased R^2^	t
Vigor	Meaningful Work	0.626	0.825	0.681	0.681	8.95 **
	Inner Life	0.276	0.847	0.718	0.037	3.94 **
Dedication	Meaningful Work	0.671	0.798	0.637	0.637	10.17
	Sense of Community	0.212	0.816	0.666	0.029	3.21
Absorption	Meaningful Work	0.586	0.679	0.461	0.461	7.39 **
	Efficacy	0.167	0.693	0.480	0.019	2.1 *
Total Scale of Work Engagement	Total Scale of Workplace Spirituality	0.502	0.800	0.640	0.640	6.70 **
	Total Scale of Psychological Capital	0.396	0.841	0.703	0.068	5.23 **

Note. Multiple R and Multiple R2 (cumulative values). * *p* < 0.05; ** *p* < 0.01.

## Data Availability

The data can be made available upon request.
